# PP38 Bevacizumab Biosimilar For Patients With Non-Small Cell Lung Cancer Or Metastatic Colorectal Cancer: Results From A Cochrane Review

**DOI:** 10.1017/S0266462325102572

**Published:** 2025-12-29

**Authors:** Annemeri Livinalli, Tais F. Galvao, Luciane C. Lopes, Ivan Zimmermann, Marcus T. Silva

## Abstract

**Introduction:**

Bevacizumab combined with chemotherapy is used in the first-line treatment of advanced non-small cell lung and metastatic colorectal cancer. Bevacizumab biosimilars have been developed and marketed after the expiration of the reference bevacizumab patent. Equivalence studies have demonstrated that biosimilars are comparable with their reference medicine, but synthesis of the effects and safety is not available to improve clinical decision-making.

**Methods:**

We performed a Cochrane systematic review and searched main bibliographic (CENTRAL, MEDLINE, Embase, Web of Science) and clinical trials databases up to February 2024. We included randomized controlled trials that directly compared adults with non-small cell lung or colorectal cancer receiving either bevacizumab biosimilars or the reference product. When applicable, we combined the results for outcomes using random-effects meta-analyses, including progression-free survival, duration of response, overall survival, mortality, objective response rate, and serious adverse events. We applied the revised Cochrane risk-of-bias tool. To assess the certainty of evidence for critical and important outcomes, we utilized the GRADE approach.

**Results:**

We included 23 studies (n=10,639 participants). Most had a high risk of bias due to issues with discontinuation and patient selection. The bevacizumab biosimilar was likely similar to the reference product in progression-free survival (hazard ratio [HR] 1.00, 95% confidence interval [CI]: 0.91, 1.09; 5 studies, n=2,660), duration of response (HR 1.05, 95% CI: 0.81, 1.37; 1 study, n=762), overall survival (HR 1.06, 95% CI: 0.94, 1.19; 5 studies, n=2,783), mortality (RR 1.03, 95% CI: 0.97, 1.09; 19 studies, n=9,231), objective response (risk ratio [RR] 0.96, 95% CI: 0.93, 1.00; 23 studies, n=10,054), and serious adverse events (RR 0.98, 95% CI: 0.93, 1.03; 23 studies, n=10,619). The certainty of evidence for all outcomes was moderate, mainly due to imprecision.

**Conclusions:**

Moderate certainty evidence indicated that bevacizumab biosimilars are likely similar to the reference biologic drug in terms of efficacy and safety.

